# Scaffold-based biomaterials in ovarian tissue engineering

**DOI:** 10.1039/d6ra00380j

**Published:** 2026-02-27

**Authors:** Chiara Di Berardino, Liliana Liverani, Aldo R. Boccaccini, Camila Cecilia Rojo-Fleming, Gianna Sacchetti, Alessia Peserico, Chiara Camerano Spelta Rapini, Giulia Capacchietti, Barbara Barboni

**Affiliations:** a Department of Bioscience and Technology for Food, Agriculture and Environment, University of Teramo 64100 Teramo Italy cdiberardino@unite.it; b Institute of Biomaterials, Department of Materials Science and Engineering, Friedrich-Alexander University of Erlangen-Nuremberg 91054 Erlangen Germany; c DGS S.p.A. 00142 Rome Italy

## Abstract

The rapid evolution of reproductive tissue engineering has positioned biomaterials as key enabling tools for the development of artificial ovary technologies aimed at fertility preservation and ovarian function restoration. Conventional assisted reproductive technologies mainly target late-stage folliculogenesis and remain inadequate for conditions involving early follicle depletion, such as gonadotoxic cancer therapies and premature ovarian insufficiency. This systematic review, conducted in accordance with PRISMA 2020 guidelines, critically examines biomimetic scaffold strategies for ovarian tissue engineering, focusing on material composition, structural design, biofunctionality and translational relevance. A total of 137 studies were analysed, covering *in vitro* and *in vivo* applications of gel-like hydrogels, decellularized extracellular matrix-derived scaffolds, electrospun fibrous constructs and 3D-printed architectures. Natural and ECM-based materials provide tissue-specific biological cues but are limited by variability and mechanical instability, whereas synthetic scaffolds, particularly electrospun poly(ε-caprolactone), offer superior control over architecture, reproducibility and scalability, supporting follicle survival across multiple mammalian models. Overall, hybrid biomaterial strategies integrating biological activity with engineered scaffold tunability emerge as the most promising platforms for artificial ovary development, while standardization and translational validation remain key challenges.

## Abstract 2

The rapid evolution of reproductive tissue engineering has positioned biomaterials as key enabling tools for the development of artificial ovary technologies aimed at fertility preservation and ovarian function restoration.

## Graphical abstract

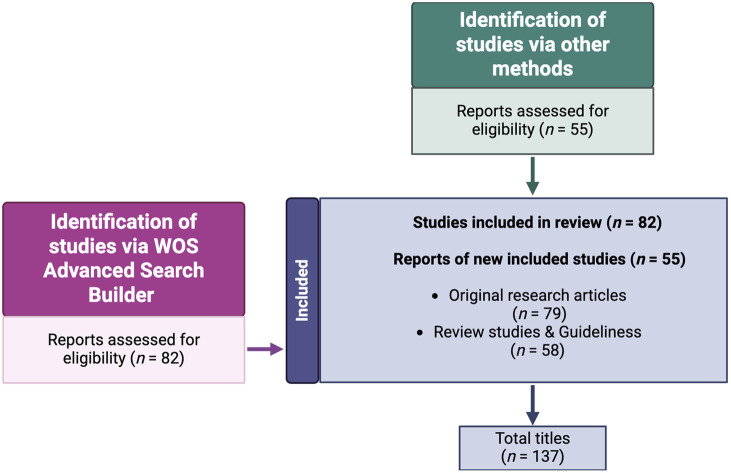

## Introduction

Regenerative medicine has become a central driver in the development of novel strategies for female fertility preservation, particularly in clinical contexts^1,2^ such as cancer treatment and premature ovarian insufficiency (POI). Gonadotoxic therapies frequently result in irreversible follicular depletion, while POI leads to an early loss of ovarian function with profound consequences for fertility and endocrine health.^3–9^ Although assisted reproductive technologies (ART) have significantly advanced reproductive care, their current applications mainly focus on late-stage folliculogenesis and therefore fail to address conditions involving depletion or dysfunction of the primordial and preantral follicle pool.^3,5,10–21^ In this context, the development of an artificial ovary represents a promising regenerative approach, aiming to recreate the ovarian niche ex vivo by integrating follicles, stromal cells, and biomaterial scaffolds into a functional three-dimensional construct.^1,22^ The success of this strategy relies on the ability to reproduce key features of the native ovarian microenvironment, including structural support, mechanical properties, and biochemical signaling essential for follicle survival and maturation.^23–27^ Recent advances in biomaterials and reproductive tissue engineering have enabled the design of biomimetic scaffolds capable of recapitulating the architecture and functionality of the ovarian extracellular matrix (ECM).^21,28,29^ These scaffolds provide a three-dimensional framework that supports follicular organization, diffusion of nutrients and hormones, and cell–matrix interactions, thereby promoting folliculogenesis and oocyte development *in vitro*.^30–32^ Despite promising progress in animal models, the translation to human clinical applications remains limited and requires further optimization of scaffold materials, structural properties and long-term biocompatibility.^21,28,33–35^

Building on these premises, this systematic review specifically focuses on the emerging and critical contribution of reproductive biomaterials to ovarian-related technologies. Expanding upon previous reviews that have highlighted 3D modeling and engineering approaches, this work offers a comprehensive overview of how biomaterial properties, biofunctionality, and translational potential collectively drive innovation in ART and clinical fertility restoration.

## Material and methods

### Bibliographic search methods

The present systematic review was carried out following the Preferred Reporting Items for Systematic Review and meta-analysis (PRISMA) Statement 2020 Checklist Guidelines (http://www.prisma-statement.org/).

Scientific literature published in the Advanced Search of Web of Science [v.5.35] “Core collection” archive (https://apps.webofknowledge.com/WOS_AdvancedSearch) was considered.

“TS” was used as a Field tag, “AND,” “OR,” and “NOT” were used as Boolean operators.

The keywords were combined to elaborate the main paragraphs, as follow:

((((((TS = (biomaterials)) AND TS = (regenerative medicine)) AND TS = (ovarian tissue engineering)) AND TS = (fertility preservation)) OR TS = (*in vitro* folliculogenesis)) AND TS = (mammal*)) NOT TS = (male).

(((((TS = (tissue engineering)) AND TS = (reproten)) AND TS = (ovarian folliculogenesis)) OR TS = (follicle growth)) AND TS = (mammal*)) AND TS = (reproduction).

((((TS = (*in vitro* follicle culture)) AND TS = (assisted reproductive technologies)) OR TS = (ART)) AND TS = (ovarian matrix))

((((((((TS = (scaffold biocompatibility)) OR TS = (fertility preservation)) AND TS = (biomimetic scaffold*)) OR TS = (electrospinning)) AND TS = (ovarian matrix)) AND TS = (reproductive health)) OR TS = (reproduction)) AND TS = (ovarian follicle)) AND TS = (reproductive tissue engineering).

### Eligibility criteria

This review focuses on exploring advancements in regenerative medicine, particularly in ovarian tissue engineering, fertility preservation, and ovarian folliculogenesis and oogenesis. Special attention was given to studies investigating innovative biomimetic strategies in reproductive tissue engineering, including the development of biomimetic scaffolds, engineered structures designed to replicate the biochemical composition, architecture, and functional cues of native extracellular matrices, thereby supporting cell adhesion, proliferation, and tissue regeneration.^36^

Only peer-reviewed, English-language publications involving mammalian models published between 1971 and 2025 were considered. Articles lacking a focus on ovarian folliculogenesis, reproductive tissue engineering, or biomimetic scaffolds were excluded. Studies on male reproductive models or non-reproductive biomedical applications were selectively included when offering valuable comparative insights into scaffold design, biocompatibility, or fabrication techniques. This strategy facilitated a comprehensive understanding of how biomimetic scaffolds can be optimized for female reproductive health applications.

### Study selection

A systematic search using the defined keywords identified 686 titles. Following the removal of duplicates, studies underwent an initial screening based on their titles and abstracts. Research emphasizing the use of biomimetic scaffolds, advanced fabrication techniques like electrospinning and 3D printing, and their role in mimicking the ovarian microenvironment for follicle growth were prioritized. The final selection included articles demonstrating scaffold biocompatibility, their application in fertility preservation, and their potential to enhance ovarian folliculogenesis. Comprehensive evaluation of full manuscripts ensured the inclusion of studies that addressed scaffold performance in reproductive tissue engineering, follicular development, and potential clinical applications. This meticulous approach ensures a focused discussion on cutting-edge advancements in regenerative medicine for reproductive health.

## Results

**Fig. 1:**
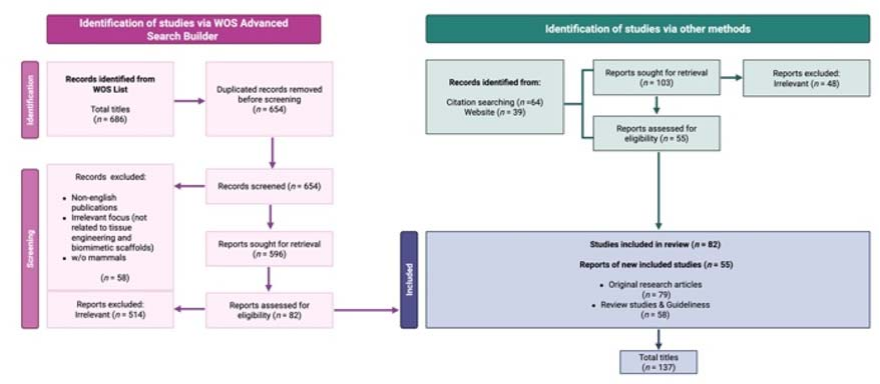
PRISMA flow diagram. The diagram shows the systematic process adopted to include papers captured by the literature search. Preferred Reporting Items for Systematic Review and *meta*-analysis” (PRISMA) Statement 2020 Checklist Guidelines were followed. Image created with https://www.Biorender.com.

A total of 137 publications met the inclusion criteria for this review, concentrating on the role of advanced scaffold technologies and materials in ovarian tissue engineering and regenerative medicine. The final selection comprised 79 original research articles, evaluated for their contributions to advancing the development and application of bioengineered scaffolds in biomedical field. Review articles and evidence-based clinical guidelines (*n* = 58) were included to provide a broader context, offering insights into the current state of ovarian tissue engineering and its clinical potential. Reference lists from these reviews were meticulously analyzed, supplemented by additional database searches, to identify further studies and significant advancements. The systematic approach adhered to the PRISMA Statement 2020 Checklist Guidelines, ensuring rigor and transparency throughout the review process. This selection highlights the multidisciplinary advancements in biomimetic scaffold design and their impact on ovarian folliculogenesis, as well as the broader implications for reproductive and non-reproductive tissue engineering (Fig. 1).

### Chemistry-driven design principles of biomaterials for ovarian tissue engineering

Recent advances in ovarian tissue engineering have highlighted that scaffold performance is not only determined by material type, but fundamentally governed by the underlying polymer chemistry, crosslinking mechanisms, and functionalization strategies. A chemistry-oriented framework enables a more rational comparison of biomaterial systems by linking molecular design to emergent structure–property–function relationships that regulate follicle survival and maturation.

### Crosslinking chemistry as a determinant of scaffold performance

Different ovarian scaffolds rely on distinct crosslinking chemistries that directly influence mechanical stiffness, degradation kinetics and diffusion properties,^37^ all of which are critical for folliculogenesis. Enzymatically polymerized fibrin hydrogels are formed through thrombin-mediated cleavage of fibrinogen, producing a covalently crosslinked fibrillar network characterized by high bioactivity but rapid proteolytic degradation.^38^ In contrast, alginate matrices undergo ionic crosslinking through divalent cations (*e.g.*, Ca^2+^), generating reversible “egg-box” junctions that confer tunable stiffness but limited cell-adhesive moieties.^39^

Synthetic PEG-based hydrogels provide an even higher degree of chemical control, as their networks can be formed *via* thiol–ene click reactions or thiol-vinyl sulfone Michael-type additions, allowing precise modulation of crosslink density and mesh size.^40,41^ This tunability directly regulates the mechanical microenvironment perceived by encapsulated follicles, which has been shown to influence activation of primordial follicles and steroidogenic activity. Collagen-based matrices, on the other hand, rely on a combination of physical self-assembly and chemical crosslinking (*e.g.*, 1-ethyl-3-(3-dimethylaminopropyl)carbodiimide/*N* hydroxysuccinimide (EDC/NHS) coupling), enabling improved mechanical stability while preserving native bioactive domains.^42,43^

### Chemical functionalization and bioactive signaling

Beyond network formation, chemical functionalization strategies play a key role in directing cell–matrix interactions.^44^ The introduction of peptide ligands such as RGD motifs, immobilization of growth factors, or incorporation of ECM-derived fragments allows the otherwise inert polymeric matrices to actively regulate granulosa cell adhesion, proliferation and paracrine signaling.^45,46^ In electrospun poly(ε-caprolactone) (PCL) scaffolds, surface modification *via* plasma treatment, hydrolysis, or grafting of bioactive molecules enhances hydrophilicity and protein adsorption, thereby compensating for the intrinsic bioinert nature of aliphatic polyesters.^47,48^ These chemical modifications highlight that scaffold biofunctionality is not merely a consequence of material origin (natural *vs.* synthetic), but rather of the density, spatial distribution and stability of chemical cues presented at the biomaterial interface.

### Structure–property-function relationships governing folliculogenesis

A unifying chemical perspective emerges when correlating molecular network design with biological outcomes. Increased crosslink density generally leads to higher elastic modulus and reduced mesh size,^49^ which can restrict follicle expansion but maintain spherical morphology and prevent premature follicle activation. Conversely, lower crosslink density results in softer matrices that promote follicle growth but may compromise structural integrity and long-term culture stability.^50^

Similarly, hydrolytic and enzymatic degradation rates, dictated by polymer backbone chemistry (*e.g.*, ester bonds in PCL *vs.* peptide bonds in fibrin), must be synchronized with the temporal dynamics of follicle maturation to ensure sustained mechanical support while allowing gradual matrix remodeling.^51^ Hydrophilicity and network porosity further regulate the diffusion of hormones, nutrients and oxygen, thereby directly impacting oocyte developmental competence.^52^

Overall, these observations demonstrate that the success of ovarian tissue engineering platforms is fundamentally rooted in the chemical design of biomaterials. A chemistry-driven classification based on crosslinking mechanisms, functionalization approaches and resulting structure–property–function relationships provide a more predictive framework than a simple categorization by material type and facilitates the rational optimization of next-generation artificial ovary scaffolds.

Based on these chemistry-driven design principles, the following sections critically analyse ovarian tissue engineering scaffolds according to their polymer chemistry, crosslinking mechanisms and resulting structure–property–function relationships.

## Reproductive tissue engineering as a new frontier for early-stage rescue

### Bioengineering strategies to promote ovarian follicle growth: the role of biomimetic scaffolds

The reproductive biotechnology community is increasingly focusing on tissue engineering advances^22,53^ driven by the synergistic integration of cutting-edge reproductive biology and engineering techniques for fabricating customized biomaterials and native matrices.^20,54^ These innovations aim to enhance protocols for both *in vivo* ovarian transplantation and *in vitro* folliculogenesis. A key enabler of this progress is the development of tissue-specific scaffolds, which form the foundation of 3D-assisted reproductive technologies (ARTs) by closely replicating the three-dimensional ovarian microenvironment (REPROTEN: REPROductive Tissue ENgineering). Within this context, biomimetic scaffolds are designed to reproduce the essential structural, mechanical, and biochemical cues of the native ovarian niche, such as appropriate matrix stiffness, nanoscale architecture, and the controlled presentation of signaling molecules, thereby fostering follicle survival, differentiation, and oocyte maturation. These features are indispensable for true biomimesis, as they determine how closely the engineered scaffold can emulate the natural extracellular matrix and support physiologically relevant cellular interactions.^36^

Scaffolds are designed to mimic the native ovarian architecture, providing a biocompatible framework that encapsulates isolated follicles and autologous ovarian cells, whose co-presence is crucial for follicular survival and functional development.^55^ Several studies have demonstrated the feasibility of these approaches, reporting viable births in mice from biomaterial implants containing isolated follicles or whole ovarian tissue.^56–58^ However, translating these findings to larger animal models or humans presents additional challenges, particularly the need to accommodate a significantly larger pool of follicles within the biomaterial constructs.^59^ Although these technologies are promising, replicating the complex *in vivo* ovarian environment remains a significant hurdle. Consequently, current advancements are predominantly at the preclinical stage, serving as a proof of concept while underscoring the potential for future clinical applications.^59^ However, scaffolds that achieve these biological aims have been developed and studied, and they can be classified in four main typologies:

#### Chemically crosslinked hydrogel networks for ovarian tissue engineering: gel-like scaffolds

Gel-like scaffold systems used in ovarian tissue engineering are fundamentally defined by their underlying crosslinking chemistry, which governs network architecture, mechanical stiffness, degradation kinetics and molecular diffusion. These systems, whether based on natural or synthetic polymers, form three-dimensional hydrogel networks through enzymatic, ionic or covalent crosslinking mechanisms. Such molecular–level interactions directly regulate follicle encapsulation, expansion and survival by modulating the biophysical and biochemical properties of the ovarian microenvironment. In this context, gel-like scaffolds are typically composed of natural or synthetic polymers, including fibrin, plasma clots, alginate-based composites such as Alginate–Nanohydroxyapatite–Collagen (ALG), polyethylene glycol (PEG) and collagen.^60^ Additional examples comprise hydrogels derived from natural biopolymers, such as hyaluronan^61^ as well as hybrid formulations incorporating decellularized matrix components, for instance Wharton's jelly/alginate composites,^62^ which further enrich the chemical and biological complexity of the resulting polymer network.

#### Composition-driven biochemical signaling in decellularized matrices: ECM-derived scaffolds

ECM-derived scaffolds represent compositionally complex biomaterials in which biological function is intrinsically linked to molecular composition and preserved biochemical motifs. The retention of collagen, laminin, elastin and glycosaminoglycan networks provides a chemically defined microenvironment capable of presenting native adhesion ligands and growth factor binding domains, thereby influencing granulosa cell behaviour, follicle survival and endocrine activity through composition-driven biochemical signaling.^63^

#### Polymer chemistry and structure–property relationships in fibrous biomaterials: electrospun scaffolds

Electrospun scaffolds are synthetic fibrous biomaterials whose performance is primarily dictated by polymer chemistry, molecular weight distribution and solvent–polymer interactions during fiber formation. These parameters determine fiber diameter, crystallinity, surface chemistry and mechanical anisotropy, which collectively define the structure–property relationships regulating follicle adhesion, spatial organization and long-term developmental competence within engineered ovarian constructs. From a structural perspective, electrospun scaffolds are characterized by a high-resolution fibrous architecture that closely mimics the nanoscale organization of the native ovarian extracellular matrix, thereby promoting cell adhesion, proliferation and follicular niche organization. These scaffolds are typically composed of biodegradable synthetic polymers, most prominently poly(ε-caprolactone) (PCL), whose tunable mechanical properties, degradation behaviour and capacity for bioactive molecule incorporation make them particularly suitable for reproductive tissue engineering and ovarian construct regeneration.^64,65^

#### Chemistry-enabled architectural and mechanical tunability: 3D-printed scaffolds

In 3D-printed ovarian scaffolds, the final architectural precision and mechanical behaviour are intrinsically controlled by the chemistry of the printable biomaterial inks, including polymer chain composition, crosslinking reactivity and rheological properties. The interplay between chemical formulation and additive manufacturing parameters enables the generation of hierarchically porous structures with tunable stiffness and degradation profiles, which are critical determinants of vascularization, nutrient diffusion and follicular niche stability after transplantation.^59^

### Efficient biomaterials for reproductive tissue engineering: biomaterial-based artificial ovary

Building upon the general classification of scaffold types discussed above, the following section provides a more detailed analysis of representative literature examples focusing on biomaterial-based artificial ovary constructs.

Reproductive system disease is a condition that affects either the male or female reproductive systems and causes problems with reproductive functions such as sexual dysfunction, subfertility, or infertility.^66^ Many therapeutic options have been explored to address this condition, including hormone therapy, medicine, and surgery. Despite significant breakthroughs, these techniques are only palliative in nature and do not cure. Many clinicians and researchers have used tissue engineering technology to restore reproductive functions in recent years.^66^ Natural polymer biomaterials, synthetic polymers, and decellularized tissue matrices have all been employed extensively in the creation of tissue engineering scaffolds.^2,65^ For the replacement of normal tissues, an ideal biomaterial should be non-inflammatory and biodegradable. Furthermore, the biomaterial should promote cell adhesion, proliferation, migration, and differentiation to enable normal tissue creation. As a result, biomaterial-based synthetic tissues may provide a feasible therapeutic option in the future for patients who require tissue replacement or repair. The artificial ovary was built using a variety of natural and synthetic gel-like matrices, including fibrin, plasma clots, Alginate-Nanohydroxyapatite-Collagen (ALG), Polyethylene glycol (PEG) and collagen, as well as ECM-based biomaterials, electrospun-based fibers and 3D-Printed scaffolds. More in detail:

#### Gel-like matrices

##### Fibrin

Because of its low inflammation, high compatibility, and good degradability, fibrin has been widely used as a bioadhesive in tissue engineering.^60,67,68^ Fibrin scaffolds were used to culture preantral (PA) follicles from both fresh and vitrified-warmed ovaries in mice. As a result, fibrinogen is frequently combined with other ingredients for follicular culture. A prior work encapsulated caprine PA follicles in fibrin-ALG hybrids.^69,70^ Fibrin-ALG may help follicle development, boost steroidogenesis, improve oocyte maturation, and produce parthenogenesis.^71^ The combination of fibrinogen and thrombin has been utilized to create autografts from isolated murine PA follicles that could survive and grow to the antral stage after only a few weeks of transplantation.^72^ Furthermore, isolated human follicles from fresh or frozen ovarian tissues were encapsulated in fibrin clots, and after 7 days of xenotransplantation, nearly 100% of the follicles were viable.^73,74^

##### Plasma clots

Plasma clots are well-known as an autologous material with the potential to sustain cell survival, stimulate cell adhesion, and proliferation, making them ideal for autologous transplantation. Gosden *et al.* initially grafted primordial follicles into plasma clots and then transplanted pups. In this investigation, ovarian cells (stromal cells and follicles included) were cultured in plasma clots before being grafted onto sterile mice. Follicular development and growth were manipulated, and healthy offspring were generated.^75^ Gosden and colleagues later discovered that mouse primordial follicles could develop in plasma clots and produce progeny following transplantation. PA follicles encased in plasma clots in humans survived and grew to the antral follicular stage after 5 months of transplantation.^76,77^ However, certain challenges remain for the use of blood clots in follicular culture and the fabrication of prosthetic ovaries, such as uneven composition and quick deterioration of such natural materials.

##### ALG

It is employed not only to encapsulate single follicles produced *in vitro*, but also to manufacture artificial ovaries. ALG is frequently utilized in conjunction with other materials such as Matrigel and poly lactic-*co*-glycolic acid (PLGA) to increase material qualities when used for artificial ovaries. Vanacker *et al.*, for example, used the ALG-Matrigel matrix to encapsulate isolated ovarian cells and follicles. The survival, proliferation, and capillary development of ovarian cells in the ALG-Matrigel were observed after 1 week of *in vitro* culture or direct autotransplantation.^78^ In following studies, ALG was utilized to encapsulate PA follicles, which were then implanted into the peritoneal pocket. About 20% of follicles formed one week after implantation, and the ALG matrix was penetrated by cells.^79^

##### PEG

PEG-based hydrogels exhibit tunable mechanical properties and high biocompatibility, making them ideal for encapsulating ovarian cells. Research by Kim *et al.* (2016) demonstrated that PEG-vinyl-sulfone (PEG-VS) support the regeneration and function of ovarian tissue in mice, highlighting their role in restoring endocrine activity and follicle viability. Despite these advantages, challenges remain in ensuring mechanical stability and *in vivo* optimization for clinical applications. Current efforts focus on integrating growth factors and improving scaffold degradation rates to match follicular maturation cycles.^80^

##### Collagen

Collagen is a widely studied natural biomaterial known for its biocompatibility and ability to promote cell adhesion. Telfer *et al.* (1990) reported that collagen-based scaffolds supported PA follicle growth and survival after transplantation under the kidney capsule in mice. Collagen enhances vascularization and nutrient delivery, which are crucial for follicle development. However, rapid degradation necessitates combination with synthetic materials to improve mechanical strength and scaffold longevity. Advances in cross-linking techniques aim to enhance collagen's stability while preserving its bioactivity.^81^

##### Hyaluronan hydrogel (HA-gel)

The HA-gel is based on hyaluronic acid, a natural glycosaminoglycan abundant in the extracellular matrix, chemically modified through tyramine linkage to enable enzymatic crosslinking and stable gel formation. In the study reported by Desai and colleagues, pre-antral mouse follicles were encapsulated within the HA-gel and cultured for 10–12 days in a three-dimensional (3D) environment.^61^ This system successfully supported antral follicle growth and oocyte maturation to the metaphase II stage following hCG stimulation.^82^ The hydrogel's viscoelastic properties were tunable by adjusting polymer concentration, allowing fine control over matrix rigidity and moldability to better reproduce physiological conditions. Furthermore, its ability to retain growth factors near the follicular cells enhanced follicle survival and functional development. Overall, HA-based hydrogels represent a promising class of gel-like biomimetic scaffolds for ovarian tissue engineering, providing a more physiologically relevant alternative to conventional two-dimensional culture systems.^61^

##### Wharton's jelly/alginate hydrogel (dWJ/Alg)

The dWJ/Alg combines decellularized Wharton's Jelly (dWJ), a matrix rich in collagen, proteoglycans, glycosaminoglycans, and growth factors, with alginate to form a hybrid gel designed to replicate the ovarian microenvironment. In the study of Tajbakhsh *et al.*, approximately 20 isolated human ovarian follicles were embedded within dWJ/Alg constructs and xenografted into ovariectomized mice.^62^ Histological analyses after one week revealed proliferation of granulosa cells and follicle growth within the dWJ/Alg implants, while control grafts composed of alginate alone exhibited markedly reduced viability and structural organization. Although complete follicle maturation was not consistently achieved, the presence of human ovarian-like tissue structures and proliferative cell populations indicated a favorable niche for folliculogenesis. The authors highlighted the potential of this composite hydrogel as a safe and functional “artificial ovary” model for fertility preservation, particularly in patients at risk of malignant cell reintroduction through ovarian tissue transplantation.^62^

#### ECM-based biomaterials

##### Decellularized ovarian ECM

ECM-based biomaterials have received a lot of interest and made significant progress in recent years.^83,84^ ECM is frequently formed during the decellularization of tissues and is mostly composed of non-water-soluble structural proteins such as collagens, laminins, and elastin.^85^ Furthermore, ECM may contain bioactive chemicals that promote cell adhesion proliferation, tissue creation, and regeneration. ECM-based biomaterials have been used successfully in clinical settings for many years by capitalizing on these features. Several investigations have been conducted in recent years on the construction of ECM-derived artificial ovaries. In a prior study, a decellularized ovarian scaffold was created from human ovaries and demonstrated good biocompatibility *in vitro.* More importantly, in the decellularized ovarian ECM, primary, primordial, or follicle-like structures could be formed, and blood estrogen and progesterone levels were significantly enhanced following transplantation.^86^ Furthermore, when ovarian cells were seeded onto decellularized ovarian scaffolds obtained from bovines or humans, they were able to create estradiol *in vitro*.^87^ Porcine ovarian tissue was decellularized and seeded with rat granulosa cells in a decellularization technique for xenogeneic ovary regeneration. The decellularized ovary tissue successfully maintained ovarian granulosa cell growth and allowed for an increase in estradiol output.^88^ Another work reseeded mouse and human ovarian cells and follicles in decellularized human ovarian tissues. The acellular scaffold demonstrated good biocompatibility, suggesting that it could aid in the survival and proliferation of human and mouse follicles.^89^ These findings indicate that decellularized ovarian ECM is a suitable biomaterial for future human ovary transplant reconstruction.

More recently, a systematic methodological review^63^ examined decellularized ECM-based scaffolds for artificial ovary construction and confirmed that preclinical studies have demonstrated support for ovarian somatic cell and follicle growth both *in vitro* and *in vivo*, though translation remains experimental. These combined findings indicate that decellularized ovarian ECM is a highly promising biomaterial for future human ovary-transplant reconstruction; however, standardization of decellularization protocols, scaffold characterization, and long-term functional verification remain key future challenges.

#### Electrospun-based fibers

##### PCL

The prevalent use of PCL in electrospun ovarian scaffolds reflects its slow hydrolytic degradation, mechanical stability and suitability for supporting the prolonged timescale required for folliculogenesis, whereas other biodegradable polyesters (*e.g.*, poly(lactic acid)/PLA, poly(lactic-*co*-glycolic acid)/PLGA) have been comparatively less explored in this specific context.

Among synthetic biomaterials, electrospun PCL has therefore emerged as a particularly promising candidate due to its excellent ability to mimic the nanofibrillar architecture of the native ECM, including that of ovarian tissue. Electrospinning is a widely adopted technique for generating scaffolds that replicate the nanoscale organization of the ECM, providing a biomimetic environment conducive to tissue regeneration.^90^ PCL is a biodegradable and biocompatible polymer that has demonstrated significant success both *in vivo* and *in vitro* across a wide range of tissues, including bone,^91–95^ cartilage,^96–100^ osteochondral,^101–105^ skin,^106–110^ nerve,^111–114^ cardiovascular,^115,116^ musculoskeletal,^117,118^ liver,^119–121^ and dental^122–128^ tissue regeneration. PCL electrospun scaffold technology has recently been applied to the construction of tailored reproductive materials, even if only a few groups of researchers are now focusing their efforts on this area, marking a significant innovation in the field.^30,64,129^ Recent researches have documented the durability of poly (epsilon-caprolactone) (PCL)-based electrospun patterned scaffolds as support for ovarian follicle growth, vitality, and retention of the fibrillary morphology of the native follicular unit.^64,129–131^ Although PCL-based scaffolds have been shown to promote porcine^130^ and ovine follicle survival,^132–134^ supporting the proof of concept of their use to generate artificial ovaries for transplantation, it is interesting to further investigated the potential of PCL-based fibers in the context of REPROTEN and more data on the ability of PCL-electrospun scaffolds to support the next steps of *in vitro* follicle/oocyte development need to be collected.

#### 3D-printed scaffolds

Complementing the electrospinning approach, 3D printing technologies offer a high degree of control over scaffold architecture, allowing precise replication of the ovarian ECM's porosity, geometry, and mechanical properties. This method enables the fabrication of complex, biomimetic designs tailored to support specific cellular and tissue requirements. Among these, gelatin-based microporous scaffolds have shown particular promise. As reported by Laronda *et al.* (2017), such scaffolds-engineered with interconnected pores-facilitate optimal nutrient and oxygen diffusion, angiogenesis, and mechanical stability, all while maintaining biodegradability and biocompatibility. These scaffolds effectively supported follicular development, endocrine activity, and fertility restoration in sterilized murine models, highlighting their therapeutic potential in ovarian regeneration. Their ability to integrate with host tissues while minimizing inflammatory responses further underscores their suitability for clinical translation. Current research is actively focused on optimizing pore design, incorporating bioactive cues, and adapting these platforms for human applications, aiming to advance artificial ovary development and reproductive tissue engineering.^59^

## Conclusions

### Concluding perspectives: comparative evaluation of biomaterials for artificial ovary engineering

A critical comparison of the biomaterials currently employed for artificial ovary engineering highlights that each scaffold class presents distinct strengths and limitations. Gel-like matrices and decellularized ECM scaffolds offer high biocompatibility and biological relevance, due to their native-like composition and ability to support cellular interactions.^60^ However, their limited mechanical stability, batch variability and lack of standardization still constrain their reproducibility and scalability for clinical applications.^63,80^

In contrast, synthetic scaffolds, particularly electrospun PCL, provide superior control over architecture, mechanical properties and degradation kinetics. Their nanofibrous structure closely resembles the ovarian extracellular matrix and has been shown to support long-term survival and development of ovarian follicles in multiple animal models.^64,129,130,132–134^ These characteristics make PCL-based scaffolds particularly attractive for the construction of functional and reproducible artificial ovaries, especially when combined with bioactive molecules or ECM-derived components to enhance biofunctionality.^90,131^

Meanwhile, 3D-printed scaffolds offer precise spatial control over pore geometry and tissue architecture, which is crucial for vascularization and integration after transplantation.^59^ However, optimization is still required to improve their mechanical stability and applicability at human scale.^59^ From a quantitative standpoint, the main classes of biomaterials employed for ovarian tissue engineering exhibit markedly different physicochemical properties that directly influence follicular development. Natural hydrogels such as fibrin, collagen and alginate typically display elastic moduli in the low kilopascal range (≈0.1–20 kPa), closely matching the native ovarian cortex stiffness and thus supporting follicle encapsulation and spherical growth.^49^ However, their relatively rapid enzymatic or hydrolytic degradation, often occurring within days to a few weeks, may limit long-term structural stability.^135^ In contrast, synthetic polymer-based scaffolds, particularly electrospun PCL, exhibit significantly higher mechanical strength, with moduli spanning from hundreds of kilopascals to the megapascal range, and degradation times extending over several months due to the slow hydrolysis of aliphatic polyester chains.^47^ These properties provide prolonged mechanical support and architectural integrity, which are advantageous for extended folliculogenesis but may require biofunctionalization to compensate for their limited intrinsic bioactivity. Porosity and pore interconnectivity further represent critical quantitative parameters, as highly porous (>70–90%) and interconnected structures facilitate oxygen and nutrient diffusion while allowing follicle expansion and vascular infiltration after transplantation.^52,136^ Overall, the balance between stiffness, degradation kinetics and porosity emerges as a key quantitative determinant of scaffold performance, highlighting the need for finely tuned physicochemical properties tailored to the temporal dynamics of follicle maturation.^137^ Overall, current evidence suggests that hybrid scaffold strategies, integrating the biological activity of natural matrices with the structural tunability of synthetic materials, represent the most promising path forward (a comprehensive comparative overview of scaffold properties and performance is provided in SI Tables 1 and 2). Importantly, future progress in artificial ovary engineering will increasingly depend on the rational chemical design of polymer networks and crosslinking strategies, rather than solely on material selection. Future developments should focus on multiscale scaffold design, incorporation of dynamic culture systems and long-term *in vivo* validation, in order to bridge the remaining gap between experimental artificial ovary models and clinical translation.

## Final remarks

### Assessment of study quality and methodological limitations

Although the current literature on biomaterials for artificial ovary engineering has significantly expanded over the past decade, several methodological limitations still restrict the translational strength of available evidence.

First, most proof-of-concept studies are conducted in murine models, which, while invaluable for initial validation, poorly replicate key aspects of human ovarian physiology, including follicular density, size scale, lifespan, and endocrine dynamics. The extrapolation of murine outcomes to the human ovarian environment therefore remains highly limited and inherently biased. In light of these limitations, future research should increasingly incorporate large animal models that more closely resemble human ovarian physiology. In particular, ovine and porcine models offer comparable follicle size, ovarian architecture and endocrine dynamics, making them more suitable for evaluating scaffold integration, vascularization and long-term folliculogenesis. In addition, non-human primate models, although limited by ethical and logistical constraints, may provide the most physiologically relevant platform for preclinical validation of artificial ovary constructs prior to clinical translation. Second, there is a marked lack of protocol standardization across studies. Scaffold fabrication methods (*e.g.*, electrospinning parameters, crosslinking protocols, porosity control), follicle isolation procedures, culture conditions, and outcome measures vary widely between laboratories. This methodological heterogeneity makes direct comparison of results difficult and hampers reproducibility, representing a major bottleneck for consortium-based or multicenter translation efforts.

Moreover, significant selection bias is evident in the current body of literature. Most studies rely on selected animal species, specific follicle stages, or optimized experimental conditions that may not reflect real-world clinical variability. Negative or inconclusive outcomes are likely underreported, leading to an overestimation of scaffold performance and translational readiness.

Finally, despite the increasing number of preclinical studies, there is a critical lack of clinical trials involving bioengineered ovarian constructs or scaffold-based artificial ovaries. Current conclusions on clinical feasibility are therefore largely speculative and must be interpreted cautiously.

Taken together, these limitations highlight the urgent need for standardized protocols, multi-species validation, and rigorously designed translational studies before artificial ovary technologies can progress toward routine clinical use.

### Clinical and regulatory perspectives

Despite the significant progress achieved in preclinical models, several key translational barriers still hinder the clinical implementation of biomaterial-based artificial ovaries. These include challenges related to large-scale standardized scaffold manufacturing, long-term safety and immunogenicity, reproducibility of follicle development across species, and the need to comply with stringent regulatory requirements for advanced therapy medicinal products. Addressing these interconnected biological, technological and regulatory constraints is essential to enable the successful translation of ovarian tissue engineering strategies from experimental settings to routine clinical practice. Beyond technical optimization, the clinical translation of biomaterial-based artificial ovaries faces substantial regulatory and safety challenges. From a manufacturing perspective, future artificial ovary scaffolds and biomaterials must comply with Good Manufacturing Practice (GMP) requirements, including reproducible production, strict quality control, sterility, batch traceability, and validation of fabrication processes, particularly if intended as implantable medical devices or advanced therapy medicinal products (ATMPs). Another critical issue concerns immunogenicity and long-term safety. Even biomimetic or decellularized matrices may retain residual antigens or trigger inflammatory responses. Furthermore, long-term follow-up is essential to evaluate risks such as chronic inflammation, fibrosis, scaffold degradation products, tumorigenicity, and potential reintroduction of malignant cells in oncological patients.

An additional critical aspect concerns the optimal *in vivo* persistence and resorption timeline of artificial ovary scaffolds. While excessively rapid biodegradation may lead to premature loss of structural support and impaired follicle maturation, overly slow degradation could result in prolonged foreign body response, fibrosis or mechanical mismatch with the regenerating tissue. Ideally, scaffold resorption should be temporally synchronized with the dynamics of folliculogenesis and tissue remodeling, providing sufficient mechanical and biochemical support during early follicle growth while gradually transferring functional load to the neo-formed ovarian tissue over a period of several months.

In addition, the path toward clinical application requires carefully designed first-in-human trials, with stringent inclusion criteria, long-term monitoring of endocrine function restoration, fertility outcomes, and safety endpoints. The complexity of implantable, bioengineered reproductive constructs demands multidisciplinary trial frameworks involving reproductive medicine specialists, bioengineers, oncologists, and regulatory experts. From a regulatory standpoint, artificial ovary technologies are likely to fall under complex frameworks governing tissue-engineered products and combination devices. Both the European Medicines Agency (EMA) and the U.S. Food and Drug Administration (FDA) impose rigorous evaluation pathways for such advanced therapies, including classification as tissue-engineered ATMPs (EMA) or combination products (FDA), with strict preclinical, toxicological, and clinical validation requirements.

Therefore, while the technological progress in reproductive biomaterials is highly promising, the transition from laboratory prototypes to clinical therapeutics will depend not only on biological performance, but also on successfully addressing regulatory, manufacturing, and safety challenges.

## Author contributions

CDB: data curation, formal analysis, methodology, writing – original draft, visualization; LL: conceptualization, supervision, original draft revision, biomaterial draft writing; ARB: conceptualization, formal analysis, writing – review & editing, visualization; CCRF: biological section writing – review & editing; GS: biomaterial writing – review & editing; AP: review & editing, visualization; CCSR: writing – review & editing, visualization; GC: writing – review & editing, visualization. BB: conceptualization, data curation, formal analysis, funding acquisition, methodology, supervision, writing original draft. All authors contributed to the manuscript and have read and approved the final version for submission.

## Conflicts of interest

There are no conflicts to declare.

## Supplementary Material

RA-016-D6RA00380J-s001

## Data Availability

No primary research results, software or code have been included and no new data were generated or analysed as part of this review. Supplementary information (SI): Tables 1 and 2 provide an overview of *in vivo* and *in vitro* studies using biomaterial-based scaffolds for artificial ovary reconstruction and follicle culture, summarizing scaffold types, experimental models, follicular stages, and key biological outcomes related to follicle survival, maturation, vascularization, and endocrine function. See DOI: https://doi.org/10.1039/d6ra00380j.

## References

[cit1] Gargus E. S., Rogers H. B., McKinnon K. E., Edmonds M. E., Woodruff T. K. (2020). Engineered reproductive tissues. Nat. Biomed. Eng..

[cit2] Atala A. (2012). Tissue engineering of reproductive tissues and organs. Fertil. Steril..

[cit3] De Vos M., Smitz J., Woodruff T. K. (2014). Fertility preservation in women with cancer. Lancet.

[cit4] Chen J., Torres-de la Roche L. A., Kahlert U. D., Isachenko V., Huang H., Hennefründ J. (2022). *et al.*, Artificial Ovary for Young Female Breast Cancer Patients. Front. Med..

[cit5] Oktem O., Urman B. (2010). Options of fertility preservation in female cancer patients. Obstet. Gynecol. Surv..

[cit6] Yeganeh L., Giri R., Flanagan M., Panay N., Anderson R. A., Bennie A. (2025). *et al.*, Evidence-based guideline: Premature Ovarian Insufficiency. Fertil. Steril..

[cit7] ESHRE Guideline (2016). management of women with premature ovarian insufficiency. Hum. Reprod..

[cit8] Jankowska K. (2017). Premature ovarian failure. Przegl. Menopauzalny.

[cit9] Kovanci E., Schutt A. K. (2015). Premature ovarian failure: Clinical presentation and treatment. Obstet. Gynecol Clin. North Am..

[cit10] Donnez J., Dolmans M. M. (2017). Fertility Preservation in Women. N. Engl. J. Med..

[cit11] Fisch B., Abir R. (2018). Female fertility preservation: Past, present and future. Reproduction.

[cit12] Dolmans M. M., Amorim C. A. (2019). FERTILITY PRESERVATION: Construction and use of artificial ovaries. Reproduction.

[cit13] Roness H., Meirow D. (2019). FERTILITY PRESERVATION: Follicle reserve loss in ovarian tissue transplantation. Reproduction.

[cit14] Duncan F. E., Zelinski M., Gunn A. H., Pahnke J. E., O'Neill C. L., Songsasen N. (2016). *et al.*, Ovarian tissue transport to expand access to fertility preservation: From animals to clinical practice. Reproduction.

[cit15] Cho E., Kim Y. Y., Noh K., Ku S. Y. (2019). A new possibility in fertility preservation: The artificial ovary. J. Tissue Eng. Regen. Med..

[cit16] Almeida G. H. D. R., Iglesia R. P., Rinaldi J. D. C., Murai M. K., Calomeno C. V. A. Q., Da Silva Junior L. N. (2023). *et al.*, Current Trends on Bioengineering Approaches for Ovarian Microenvironment Reconstruction. Tissue Eng. Part B Rev..

[cit17] Jones A. S. K., Shikanov A. (2019). Follicle development as an orchestrated signaling network in a 3D organoid. J. Biol. Eng..

[cit18] Jain M. and Singh M., Assisted Reproductive Technology (ART) Techniques, StatPearls, 2022, https://www.ncbi.nlm.nih.gov/books/NBK576409/35015434

[cit19] Monniaux D. (2018). Factors influencing establishment of the ovarian reserve and their effects on fertility. Anim. Reprod..

[cit20] Amorim C. A. (2017). Special Issue Devoted to a New Field of Regenerative Medicine: Reproductive Tissue Engineering. Ann. Biomed. Eng..

[cit21] Jain P., Kathuria H., Dubey N. (2022). Advances in 3D bioprinting of tissues/organs for regenerative medicine and in-vitro models. Biomaterials.

[cit22] Bakhshandeh B., Zarrintaj P., Oftadeh M. O., Keramati F., Fouladiha H., Sohrabi-jahromi S. (2017). *et al.*, Tissue engineering; strategies, tissues, and biomaterials. Biotechnol. Genet. Eng. Rev..

[cit23] Xu M., Kreeger P. K., Shea L. D., Woodruff T. K. (2006). Tissue-engineered follicles produce live, fertile offspring. Tissue Eng..

[cit24] Shea L. D., Woodruff T. K., Shikanov A. (2014). Bioengineering the ovarian follicle microenvironment. Annu. Rev. Biomed. Eng..

[cit25] Zubizarreta M. E., Xiao S. (2020). Bioengineering models of female reproduction. Biodes. Manuf..

[cit26] Xu M., Woodruff T. K., Shea L. D. (2007). Bioengineering and the ovarian follicle. Cancer Treat Res..

[cit27] Francés-Herrero E., Lopez R., Hellström M., de Miguel-Gómez L., Herraiz S., Brännström M. (2022). *et al.*, Bioengineering trends in female reproduction: a systematic review. Hum. Reprod. Update.

[cit28] Maharjan S., Ma C., Singh B., Kang H., Orive G., Yao J. (2024). *et al.*, Advanced 3D imaging and organoid bioprinting for biomedical research and therapeutic applications. Adv. Drug Deliv. Rev..

[cit29] O'Brien F. J. (2011). Biomaterials & scaffolds for tissue engineering. Mater. Today.

[cit30] Zhang T., Zhang M., Zhang S., Wang S. (2024). Research advances in the construction of stem cell-derived ovarian organoids. Stem Cell Res. Ther..

[cit31] Cabral M., Cheng K., Zhu D. (2024). Three-Dimensional Bioprinting of Organoids: Past, Present, and Prospective. Tissue Eng..

[cit32] Kalyuzhny E., Mierke C. T. (2024). Bioprinting of Cells, Organoids and Organs-on-a-Chip Together with Hydrogels Improves Structural and Mechanical Cues. Cells.

[cit33] Frankowski J., Kurzątkowska M., Sobczak M., Piotrowska U. (2023). Utilization of 3D bioprinting technology in creating human tissue and organoid models for preclinical drug research – State-of-the-art. Int. J. Pharm..

[cit34] Amorim C. A., Shikanov A. (2016). The artificial ovary: current status and future perspectives. Future Oncol..

[cit35] Chiti M. C., Donnez J., Amorim C. A., Dolmans M. M. (2018). From isolation of human ovarian follicles to the artificial ovary: tips and tricks. Minerva Obstet Gynecol..

[cit36] Baiguera S., Di Silvio L., Del Gaudio C. (2024). Moving Toward Biomimetic Tissue-Engineered Scaffolds. Nanomaterials.

[cit37] Caló E., Khutoryanskiy V. V. (2015). Biomedical applications of hydrogels: A review of patents and commercial products. Eur. Polym. J..

[cit38] Janmey P. A., Winer J. P., Weisel J. W. (2009). Fibrin gels and their clinical and bioengineering applications. J. R. Soc., Interface.

[cit39] Lee K. Y., Mooney D. J. (2012). Alginate: Properties and biomedical applications. Prog. Polym. Sci..

[cit40] McGann C. L., Dumm R. E., Jurusik A. K., Sidhu I., Kiick K. L. (2016). Thiol-ene Photocrosslinking of Cytocompatible Resilin-Like Polypeptide-PEG Hydrogels. Macromol. Biosci..

[cit41] Hoyle C. E., Bowman C. N. (2010). Thiol-ene click chemistry. Angew Chem. Int. Ed. Engl..

[cit42] Olde Damink L. H. H., Dijkstra P. J., Van Luyn M. J. A., Van Wachem P. B., Nieuwenhuis P., Feijen J. (1996). Cross-linking of dermal sheep collagen using a water-soluble carbodiimide. Biomaterials.

[cit43] Zeugolis D. I., Khew S. T., Yew E. S. Y., Ekaputra A. K., Tong Y. W., Yung L. Y. L. (2008). *et al.*, Electro-spinning of pure collagen nano-fibres - Just an expensive way to make gelatin?. Biomaterials.

[cit44] Lutolf M. P., Hubbell J. A. (2005). Synthetic biomaterials as instructive extracellular microenvironments for morphogenesis in tissue engineering. Nat. Biotechnol..

[cit45] Hersel U., Dahmen C., Kessler H. (2003). RGD modified polymers: Biomaterials for stimulated cell adhesion and beyond. Biomaterials.

[cit46] Martino M. M., Briquez P. S., Güç E., Tortelli F., Kilarski W. W., Metzger S. (2014). *et al.*, Growth factors engineered for super-affinity to the extracellular matrix enhance tissue healing. Science.

[cit47] Woodruff M. A., Hutmacher D. W. (2010). The return of a forgotten polymer—Polycaprolactone in the 21st century. Prog. Polym. Sci..

[cit48] Martins A., Pinho E. D., Faria S., Pashkuleva I., Marques A. P., Reis R. L. (2009). *et al.*, Surface modification of electrospun polycaprolactone nanofiber meshes by plasma treatment to enhance biological performance. Small.

[cit49] Caliari S. R., Burdick J. A. (2016). A practical guide to hydrogels for cell culture. Nat. Methods.

[cit50] Wells R. G. (2008). The role of matrix stiffness in regulating cell behavior. Hepatology.

[cit51] Anderson J. M., Shive M. S. (1997). Biodegradation and biocompatibility of PLA and PLGA microspheres. Adv. Drug Deliv. Rev..

[cit52] Griffith L. G., Swartz M. A. (2006). Capturing complex 3D tissue physiology in vitro. Nat. Rev. Mol. Cell Biol..

[cit53] Sharma P., Kumar P., Sharma R., Bhatt V. D., Dhot P. S. (2019). Tissue Engineering; Current Status & Futuristic Scope. J. Life Med..

[cit54] Liverani L., Guarino V., La Carrubba V., Boccaccini A. R. (2017). Porous biomaterials and scaffolds for tissue engineering. Encycl. Biomater. Biomed. Eng..

[cit55] Soares M., Sahrari K., Amorim C. A., Saussoy P., Donnez J., Dolmans M. M. (2015). Evaluation of a human ovarian follicle isolation technique to obtain disease-free follicle suspensions before safely grafting to cancer patients. Fertil. Steril..

[cit56] Shikanov A., Zhang Z., Xu M., Smith R. M., Rajan A., Woodruff T. K. (2011). *et al.*, Fibrin encapsulation and vascular endothelial growth factor delivery promotes ovarian graft survival in mice. Tissue Eng..

[cit57] Kniazeva E., Hardy A. N., Boukaidi S. A., Woodruff T. K., Jeruss J. S., Shea L. D. (2016). Primordial Follicle Transplantation within Designer Biomaterial Grafts Produce Live Births in a Mouse Infertility Model. Sci. Rep..

[cit58] Carroll J., Gosden R. G. (1993). Transplantation of frozen-thawed mouse primordial follicles. Hum. Reprod..

[cit59] Laronda M. M., Rutz A. L., Xiao S., Whelan K. A., Duncan F. E., Roth E. W. (2017). *et al.*, A bioprosthetic ovary created using 3D printed microporous scaffolds restores ovarian function in sterilized mice. Nat. Commun..

[cit60] Chiti M. C., Dolmans M. M., Donnez J., Amorim C. A. (2017). Fibrin in Reproductive Tissue Engineering: A Review on Its Application as a Biomaterial for Fertility Preservation. Ann. Biomed. Eng..

[cit61] Desai N., Attard M., Spangler M., Gishto A., Brown A., Wirtjes M. (2025). Application of a Novel Hyaluronan Hydrogel for Three-Dimensional Follicle Culture and Methodology for Mouse Ovarian Follicle Cryopreservation. J. Visualized Exp..

[cit62] Tajbakhsh F., Tavana S., Kazemi Ashtiani M., Moini A., Amorim C. A., Fathi R. (2025). Wharton's Jelly Hydrogel: An Innovative Artificial Ovary for Xenotransplantation of Isolated Human Ovarian Follicles. Biology.

[cit63] Wu T., Huang K. C., Yan J. F., Zhang J. J., Wang S. X. (2023). Extracellular matrix-derived scaffolds in constructing artificial ovaries for ovarian failure: a systematic methodological review. Hum. Reprod. Open.

[cit64] Liverani L., Raffel N., Fattahi A., Preis A., Hoffmann I., Boccaccini A. R. (2019). *et al.*, Electrospun patterned porous scaffolds for the support of ovarian follicles growth: a feasibility study. Sci. Rep..

[cit65] Tamadon A., Park K. H., Kim Y. Y., Kang B. C., Ku S. Y. (2016). Efficient biomaterials for tissue engineering of female reproductive organs. J. Tissue Eng. Regen. Med..

[cit66] Wang X., Wu D., Li W., Yang L. (2021). Emerging biomaterials for reproductive medicine. Eng. Regen..

[cit67] Rajabzadeh A. R., Eimani H., Mohseni Koochesfahani H., Shahvardi A. H., Fathi R. (2015). Morphological study of isolated ovarian preantral follicles using fibrin gel plus platelet lysate after subcutaneous transplantation. J. Cell..

[cit68] Smith R. M., Shikanov A., Kniazeva E., Ramadurai D., Woodruff T. K., Shea L. D. (2014). Fibrin-mediated delivery of an ovarian follicle pool in a mouse model of infertility. Tissue Eng..

[cit69] Shikanov A., Xu M., Woodruff T. K., Shea L. D. (2011). A Method for Ovarian Follicle Encapsulation and Culture in a Proteolytically Degradable 3 Dimensional System. J. Visualized Exp..

[cit70] Shikanov A., Xu M., Woodruff T. K., Shea L. D. (2009). Interpenetrating fibrin-alginate matrices for in vitro ovarian follicle development. Biomaterials.

[cit71] Brito I. R., Silva G. M., Sales A. D., Lobo C. H., Rodrigues G. Q., Sousa R. F. (2016). *et al.*, Fibrin-alginate hydrogel supports steroidogenesis, in vitro maturation of oocytes and parthenotes production from caprine preantral follicles cultured in group. Reprod. Domest. Anim..

[cit72] Chiti M. C., Dolmans M. M., Orellana R., Soares M., Paulini F., Donnez J. (2016). *et al.*, Influence of follicle stage on artificial ovary outcome using fibrin as a matrix. Hum. Reprod..

[cit73] Paulini F., Vilela J. M. V., Chiti M. C., Donnez J., Jadoul P., Dolmans M. M. (2016). *et al.*, Survival and growth of human preantral follicles after cryopreservation of ovarian tissue, follicle isolation and short-term xenografting. Reprod. Biomed. Online.

[cit74] Chiti M. C., Dolmans M. M., Hobeika M., Cernogoraz A., Donnez J., Amorim C. A. (2017). A modified and tailored human follicle isolation procedure improves follicle recovery and survival. J. Ovarian Res..

[cit75] Gosden R. G. (1990). Restitution of fertility in sterilized mice by transferring primordial ovarian follicles. Hum. Reprod..

[cit76] Dolmans M. M., Martinez-Madrid B., Gadisseux E., Guiot Y., Yuan W. Y., Torre A. (2007). *et al.*, Short-term transplantation of isolated human ovarian follicles and cortical tissue into nude mice. Reproduction.

[cit77] Dolmans M. M., Yuan W. Y., Camboni A., Torre A., Van Langendonckt A., Martinez-Madrid B. (2008). *et al.*, Development of antral follicles after xenografting of isolated small human preantral follicles. Reprod. Biomed. Online.

[cit78] Vanacker J., Luyckx V., Dolmans M. M., Des Rieux A., Jaeger J., Van Langendonckt A. (2012). *et al.*, Transplantation of an alginate-matrigel matrix containing isolated ovarian cells: first step in developing a biodegradable scaffold to transplant isolated preantral follicles and ovarian cells. Biomaterials.

[cit79] Vanacker J., Dolmans M. M., Luyckx V., Donnez J., Amorim C. A. (2014). First transplantation of isolated murine follicles in alginate. Regener. Med..

[cit80] Kim J., Perez A. S., Claflin J., David A., Zhou H., Shikanov A. (2016). Synthetic hydrogel supports the function and regeneration of artificial ovarian tissue in mice. NPJ Regen. Med..

[cit81] Telfer E., Torrance C., Gosden R. G. (1990). Morphological study of cultured preantral ovarian follicles of mice after transplantation under the kidney capsule. J. Reprod. Fertil..

[cit82] Desai N., Spangler M., Nanavaty V., Gishto A., Brown A. (2022). New hyaluronan-based biomatrix for 3-D follicle culture yields functionally competent oocytes. Reprod. Biol. Endocrinol..

[cit83] Mayorca-Guiliani A. E., Willacy O., Madsen C. D., Rafaeva M., Elisabeth Heumüller S., Bock F. (2019). *et al.*, Decellularization and antibody staining of mouse tissues to map native extracellular matrix structures in 3D. Nat. Protoc..

[cit84] Jakus A. E., Laronda M. M., Rashedi A. S., Robinson C. M., Lee C., Jordan S. W. (2017). *et al.*, “Tissue Papers” from Organ-Specific Decellularized Extracellular Matrices. Adv. Funct. Mater..

[cit85] Rijal G. (2017). The decellularized extracellular matrix in regenerative. Medicine.

[cit86] Hassanpour A., Talaei-Khozani T., Kargar-Abarghouei E., Razban V., Vojdani Z. (2018). Decellularized human ovarian scaffold based on a sodium lauryl ester sulfate (SLES)-treated protocol, as a natural three-dimensional scaffold for construction of bioengineered ovaries. Stem Cell Res. Ther..

[cit87] Laronda M. M., Jakus A. E., Whelan K. A., Wertheim J. A., Shah R. N., Woodruff T. K. (2015). Initiation of puberty in mice following decellularized ovary transplant. Biomaterials.

[cit88] Liu W. Y., Lin S. G., Zhuo R. Y., Xie Y. Y., Pan W., Lin X. F. (2017). *et al.*, Xenogeneic Decellularized Scaffold: A Novel Platform for Ovary Regeneration. Tissue Eng., Part C.

[cit89] Pors S. E., Ramløse M., Nikiforov D., Lundsgaard K., Cheng J., Yding A. C. (2019). *et al.*, Initial steps in reconstruction of the human ovary: survival of pre-antral stage follicles in a decellularized human ovarian scaffold. Hum. Reprod..

[cit90] Siddiqui N., Asawa S., Birru B., Baadhe R., Rao S. (2018). PCL-Based Composite Scaffold Matrices for Tissue Engineering Applications. Mol. Biotechnol..

[cit91] Kim Y. B., Kim G. H. (2015). PCL/alginate composite scaffolds for hard tissue engineering: fabrication, characterization, and cellular activities. ACS Comb. Sci..

[cit92] Rumiński S., Ostrowska B., Jaroszewicz J., Skirecki T., Włodarski K., Święszkowski W. (2018). *et al.*, Three-dimensional printed polycaprolactone-based scaffolds provide an advantageous environment for osteogenic differentiation of human adipose-derived stem cells. J. Tissue Eng. Regen. Med..

[cit93] Ronca D., Langella F., Chierchia M., D'Amora U., Russo T., Domingos M. (2016). *et al.*, Bone Tissue Engineering: 3D PCL-based Nanocomposite Scaffolds with Tailored Properties. Proced. CIRP.

[cit94] Van Rie J., Declercq H., Van Hoorick J., Dierick M., Van Hoorebeke L., Cornelissen R. (2015). *et al.*, Cryogel-PCL combination scaffolds for bone tissue repair. J. Mater. Sci. Mater. Med..

[cit95] Yoshimoto H., Shin Y. M., Terai H., Vacanti J. P. (2003). A biodegradable nanofiber scaffold by electrospinning and its potential for bone tissue engineering. Biomaterials.

[cit96] Liu J., Fang Q., Yu X., Wan Y., Xiao B. (2018). Chitosan-Based Nanofibrous Membrane Unit with Gradient Compositional and Structural Features for Mimicking Calcified Layer in Osteochondral Matrix. Int. J. Mol. Sci..

[cit97] Lee L. W., Hsiao S. H., Hung W. C., Lin Y. H., Chen P. Y., Chiang C. P. (2015). Clinical outcomes for teeth treated with electrospun poly(ε-caprolactone) fiber meshes/mineral trioxide aggregate direct pulp capping. J. Endod..

[cit98] Chang K. Y., Hung L. H., Chu I. M., Ko C. S., Lee Y. Der (2010). The application of type II collagen
and chondroitin sulfate grafted PCL porous scaffold in cartilage tissue engineering. J. Biomed. Mater. Res., Part A.

[cit99] Kim H. J., Lee J. H., Im G. Il (2010). Chondrogenesis using mesenchymal stem cells and PCL scaffolds. J. Biomed. Mater. Res., Part A.

[cit100] Valonen P. K., Moutos F. T., Kusanagi A., Moretti M. G., Diekman B. O., Welter J. F. (2010). *et al.*, In vitro generation of mechanically functional cartilage grafts based on adult human stem cells and 3D-woven poly(epsilon-caprolactone) scaffolds. Biomaterials.

[cit101] Swieszkowski W., Tuan B. H. S., Kurzydlowski K. J., Hutmacher D. W. (2007). Repair and regeneration of osteochondral defects in the articular joints. Biomol. Eng..

[cit102] Du Y., Liu H., Yang Q., Wang S., Wang J., Ma J. (2017). *et al.*, Selective laser sintering scaffold with hierarchical architecture and gradient composition for osteochondral repair in rabbits. Biomaterials.

[cit103] Mellor L. F., Huebner P., Cai S., Mohiti-Asli M., Taylor M. A., Spang J. (2017). *et al.*, Fabrication and Evaluation of Electrospun, 3D-Bioplotted, and Combination of Electrospun/3D-Bioplotted Scaffolds for Tissue Engineering Applications. BioMed Res. Int..

[cit104] Yeo M., Kim G. (2013). Cell-printed hierarchical scaffolds consisting of micro-sized polycaprolactone (PCL) and electrospun PCL nanofibers/cell-laden alginate struts for tissue regeneration. J. Mater. Chem. B.

[cit105] Hsieh Y. H., Hsieh M. F., Fang C. H., Jiang C. P., Lin B., Lee H. M. (2017). Osteochondral Regeneration Induced by TGF-β Loaded Photo Cross-Linked Hyaluronic Acid Hydrogel Infiltrated in Fused Deposition-Manufactured Composite Scaffold of Hydroxyapatite and Poly (Ethylene Glycol)-Block-Poly(ε-Caprolactone). Polymers.

[cit106] Firoozi N., Rezayan A. H., Tabatabaei Rezaei S. J., Mir-Derikvand M., Nabid M. R., Nourmohammadi J. (2017). Synthesis of poly(ε-caprolactone)-based polyurethane semi-interpenetrating polymer networks as scaffolds for skin tissue regeneration. Int. J. Polym. Mater. Polym. Biomater.

[cit107] Ghosal K., Manakhov A., Zajíčková L., Thomas S. (2017). Structural and Surface Compatibility Study of Modified Electrospun Poly(ε-caprolactone) (PCL) Composites for Skin Tissue Engineering. AAPS PharmSciTech.

[cit108] Powell H. M., Boyce S. T. (2009). Engineered human skin fabricated using electrospun collagen-PCL blends: morphogenesis and mechanical properties. Tissue Eng..

[cit109] Gautam S., Chou C. F., Dinda A. K., Potdar P. D., Mishra N. C. (2014). Surface modification of nanofibrous polycaprolactone/gelatin composite scaffold by collagen type I grafting for skin tissue engineering. Mater. Sci. Eng. C.

[cit110] Lou T., Leung M., Wang X., Chang J. Y. F., Tsao C. T., Sham J. G. C. (2014). *et al.*, Bi-layer scaffold of chitosan/PCL-nanofibrous mat and PLLA-microporous disc for skin tissue engineering. J. Biomed. Nanotechnol..

[cit111] Bolaina-Lorenzo E., Martinez-Ramos C., Monleón-Pradas M., Herrera-Kao W., Cauich-Rodriguez J. V., Cervantes-Uc J. M. (2016). Electrospun polycaprolactone/chitosan scaffolds for nerve tissue engineering: physicochemical characterization and Schwann cell biocompatibility. Biomed. Mater..

[cit112] Ghasemi-Mobarakeh L., Prabhakaran M. P., Morshed M., Nasr-Esfahani M. H., Ramakrishna S. (2010). Bio-functionalized PCL nanofibrous scaffolds for nerve tissue engineering. Mater. Sci. Eng. C.

[cit113] Mohammadi S., Shafiei S. S., Asadi-Eydivand M., Ardeshir M., Solati-Hashjin M. (2016). Graphene oxide-enriched poly(ε-caprolactone) electrospun nanocomposite scaffold for bone tissue
engineering applications. J. Bioact. Compat. Polym..

[cit114] Niu Y., Chen K. C., He T., Yu W., Huang S., Xu K. (2014). Scaffolds from block polyurethanes based on poly(ε-caprolactone) (PCL) and poly(ethylene glycol) (PEG) for peripheral nerve regeneration. Biomaterials.

[cit115] Ahmed L. A. (2013). Stem cells and cardiac repair: alternative and multifactorial approaches. J. Regen. Med. Tissue Eng..

[cit116] Baheiraei N., Yeganeh H., Ai J., Gharibi R., Ebrahimi-Barough S., Azami M. (2015). *et al.*, Preparation of a porous conductive scaffold from aniline pentamer-modified polyurethane/PCL blend for cardiac tissue engineering. J. Biomed. Mater. Res., Part A.

[cit117] Chen M. C., Sun Y. C., Chen Y. H. (2013). Electrically conductive nanofibers with highly oriented structures and their potential application in skeletal muscle tissue engineering. Acta Biomater..

[cit118] Fasolino I., Guarino V., Cirillo V., Ambrosio L. (2017). 5-Azacytidine-mediated hMSC behavior on electrospun scaffolds for skeletal muscle regeneration. J. Biomed. Mater. Res., Part A.

[cit119] Semnani D., Naghashzargar E., Hadjianfar M., Dehghan Manshadi F., Mohammadi S., Karbasi S. (2016). Evaluation of PCL/chitosan electrospun nanofibers for liver tissue engineering. Int. J. Polym. Mater. Polym. Biomater.

[cit120] Huang H., Oizumi S., Kojima N., Niino T., Sakai Y. (2007). Avidin–biotin binding-based cell seeding and perfusion culture of liver-derived cells in a porous scaffold with a three-dimensional interconnected flow-channel network. Biomaterials.

[cit121] Grant R., Hay D. C., Callanan A. (2017). A Drug-Induced Hybrid Electrospun Poly-Capro-Lactone: Cell-Derived Extracellular Matrix Scaffold for Liver Tissue Engineering. Tissue Eng..

[cit122] Zhang L., Morsi Y., Wang Y., Li Y., Ramakrishna S. (2013). Review scaffold design and stem cells for tooth regeneration. Jpn Dent. Sci Rev..

[cit123] Yang X., Yang F., Walboomers X. F., Bian Z., Fan M., Jansen J. A. (2010). The performance of dental pulp stem cells on nanofibrous PCL/gelatin/nHA scaffolds. J. Biomed. Mater. Res., Part A.

[cit124] Wu Y., Azmi D. F. B., Rosa V., Fawzy A. S., Fuh J. Y. H., Wong Y. S. (2016). *et al.*, Fabrication of dentin-like scaffolds through combined 3D printing and bio-mineralisation. Cogent Eng..

[cit125] Jensen J., Kraft D. C. E., Lysdahl H., Foldager C. B., Chen M., Kristiansen A. A. (2015). *et al.*, Functionalization of polycaprolactone scaffolds with hyaluronic acid and β-TCP facilitates migration and osteogenic differentiation of human dental pulp stem cells *in vitro*. Tissue Eng..

[cit126] Flores-Cedillo M. L., Alvarado-Estrada K. N., Pozos-Guillén A. J., Murguía-Ibarra J. S., Vidal M. A., Cervantes-Uc J. M. (2016). *et al.*, Multiwall carbon nanotubes/polycaprolactone scaffolds seeded with human dental pulp stem cells for bone tissue regeneration. J. Mater. Sci. Mater. Med..

[cit127] Wurth J. J., Blumenthal N. R., Prasad S. V. (2014). Hydrophilization of Poly(Caprolactone) Copolymers through Introduction of Oligo(Ethylene Glycol) Moieties. PLoS One.

[cit128] Khodaverdi E., Gharechahi M., Alibolandi M., Tekie F. M., Khashyarmanesh B., Hadizadeh F. (2016). Self-assembled supramolecular hydrogel based on PCL-PEG-PCL triblock copolymer and γ-cyclodextrin inclusion complex for sustained delivery of dexamethasone. Int. J. Pharm. Invest..

[cit129] Raffel N., Dittrich R., Bäuerle T., Seyler L., Fattahi A., Hoffmann I. (2019). *et al.*, Novel approach for the assessment of ovarian follicles infiltration in polymeric electrospun patterned scaffolds. PLoS One.

[cit130] Fattahi A., Liverani L., Dittrich R., Hoffmann I., Boccaccini A. R., Beckmann M. W. (2020). *et al.*, Optimization of Porcine Ovarian Follicle Isolation Methods for Better Developmental Potential. Tissue Eng..

[cit131] Liverani L., Boccaccini A. R. (2016). Versatile Production of Poly(Epsilon-Caprolactone) Fibers by Electrospinning Using Benign Solvents. Nanomaterials.

[cit132] Di Berardino C., Liverani L., Peserico A., Capacchietti G., Russo V., Bernabò N. (2022). *et al.*, When Electrospun Fiber Support Matters: In Vitro Ovine Long-Term Folliculogenesis on Poly (Epsilon Caprolactone) (PCL)-Patterned Fibers. Cells.

[cit133] Peserico A., Di Berardino C., Capacchietti G., Camerano Spelta Rapini C., Liverani L., Boccaccini A. R. (2023). *et al.*, IVM Advances for Early Antral Follicle-Enclosed Oocytes Coupling Reproductive Tissue Engineering to Inductive Influences of Human Chorionic Gonadotropin and Ovarian Surface Epithelium Coculture. Int. J. Mol. Sci..

[cit134] Di Berardino C., Peserico A., Camerano Spelta Rapini C., Liverani L., Capacchietti G., Russo V. (2024). *et al.*, Bioengineered 3D ovarian model for long-term multiple development of preantral follicle: bridging the gap for poly(ε-caprolactone) (PCL)-based scaffold reproductive applications. Reprod. Biol. Endocrinol..

[cit135] Sun J., Tan H. (2013). Alginate-Based Biomaterials for Regenerative Medicine Applications. Materials.

[cit136] Karande T. S., Ong J. L., Agrawal C. M. (2004). Diffusion in musculoskeletal tissue engineering scaffolds: design issues related to porosity, permeability, architecture, and nutrient mixing. Ann. Biomed. Eng..

[cit137] Tibbitt M. W., Anseth K. S. (2009). Hydrogels as extracellular matrix mimics for 3D cell culture. Biotechnol. Bioeng..

