# Supplementary material for: Scaffold-based biomaterials in ovarian tissue engineering
Source: RSC Adv. 2026 Feb 27;16(13):11530–42. doi: 10.1039/d6ra00380j (PMC12947750; doi:10.1039/d6ra00380j)
Supplement: RA-016-D6RA00380J-s001 [file RA-016-D6RA00380J-s001.pdf]

Supplementary Information (SI) for RSC Advances.
This journal is © The Royal Society of Chemistry 2026

**Supplementary Table 1: Overview of *in vivo* studies using biomaterial scaffolds for artificial ovary reconstruction**: summary of studies that evaluated biomaterial-based scaffolds in in vivo contexts (animal models or xenografts) for ovarian tissue engineering or artificial ovary development.

The table compares scaffold composition, applications, and biological outcomes across studies, emphasizing graft location, follicular stages involved, and combinations with other materials.

Biomaterials include fibrin, PEG, alginate, composite hydrogels, Wharton's jelly, decellularized ECM, and 3D-printed gelatin scaffolds. These approaches primarily aimed to restore endocrine and reproductive function, test biocompatibility, and support follicle survival and vascularization in mice and other species.

| *Biomaterial Class* | Material | Applications | Advantages | Disadvantages | Future Perspectives | Follicle Stage | Grafting Location | Species | Conjunctions with Other Materials | References |
|---|---|---|---|---|---|---|---|---|---|---|
| *Gel-like scaffold matrices* | **Fibrin** | Follicle encapsulation and transplantation(xenografts/allografts) | Biocompatible, low immune response, supports survival & growth | Limited stability unless combined with other polymers | Optimize blends/crosslinking for more durable grafts | Primordial & preantral | Kidney capsule, abdominal cavity, ovarian bursa | Mice, human follicles xenografted | Often with ALG; also HA | (1); (2); (3); (4) |
| | **PEG-VS (PEG hydrogel)** | Hydrogel encapsulation of ovarian follicles with in vivo function restoration | Tunable mechanics; supports vascularization and endocrine function | Needs optimization for long-term mechanical integrity | Improve composite designs & bioactivity for clinical translation | Primordial, primary, preantral | Abdominal/peritoneal pockets, ovarian bursa | Mice | With other hydrogels/ECM cues | (5) |
| | **Plasma clots** | Autologous clot as carrier for isolated follicles and ovarian cells xenografted/autografted | Autologous, supports adhesion, early vascular invasion | Fast degradation; composition not standardized | Stabilize clots or blend with fibrin/ALG for longevity | Primordial | Peritoneal pocket, ovarian bursa, intraovarian | Mice, human follicles xenografted | Standalone/autologous | (6) |
| | **Alginate (ALG)** | Beads carrying isolated follicles + ovarian cells for transplantation | Supports capillary ingrowth; versatile | Needs reinforcement/biodegradation tuning | Composite ALG matrices for safer retrieval and function | Primordial & preantral | Peritoneal/subcutaneous pockets, abdominal cavity | Mice | Matrigel, PLGA, fibrin | (7); (8) |
| | **Wharton's Jelly/Alginate (dWJ/Alg)** | Xenotransplant of human follicles into ovariectomized mice; short-term endocrine & histology readouts | Collagen/GAGs/growth factor-rich; supports GC proliferation | Short-term; inflammation noted; variability | Long-term endocrine studies; optimize ratios and biosafety | Primordial & preantral | (xenotransplant into NMRI mice; peritoneal region) | Mice, human follicles xenografted | With ALG | (9) |
| *Natural ECM-derived scaffolds* | **Decellularized ovarian ECM** | Xenotransplant-ready matrices; endocrine support | High biocompatibility; tissue-specific cues | Process standardization/scaling | Standardized decell protocols; human validation | Cortical ECM (follicle niche-derived) | Proposed heterotopic/orthotopic | Mice, porcine follicles xenografted | - | (10); (11) |
| *3D printed scaffolds* | **Microporous gelatin (3D printed)** | Bioprosthetic ovary restores ovarian function and fertility in sterilized mice | Biocompatible; robust in vivo evidence of endocrine and reproductive function | Scaling to human size/grade | Clinical-grade biomaterial + topology optimization | Primordial & preantral | Orthotopic sites | Mice | - | (12) |

***Supplementary Table 2: Overview of in vitro studies using biomaterial scaffolds for follicle culture and artificial ovary modeling***: summary of in vitro studies exploring biomaterials and engineered scaffolds to support folliculogenesis and oocyte maturation outside the body.

The table reports the biomaterial class, follicle stages cultured, key advantages and limitations, and species employed. Tested materials include fibrin, alginate, hyaluronan-based hydrogels, collagen, decellularized ovarian ECM, and electrospun PCL scaffolds.

These studies provide insight into how ECM-mimicking materials can sustain three-dimensional follicle structure, growth, and hormone production, representing essential steps toward a fully functional artificial ovary.

| ***Biomaterial Class*** | **Material** | **Applications** | **Advantages** | **Disadvantages** | **Future Perspectives** | **Follicle Stage** | **Species** | **Conjunctions with Other Materials** | **References** |
|---|---|---|---|---|---|---|---|---|---|
| *Gel-like scaffold matrices* | **Fibrin / Fibrin-Alginate (FA-IPN)** | 3D follicle culture**;** improved caprine/mouse preantral growth; steroidogenesis | ECM-like matrix; dynamic mechanics (with IPN); supports growth & E2/P4 | Pure fibrin less stable; IPN design complexity | Tuned degradability/ crosslinking for human follicles | From Primordial to antral | Caprine, mouse | With ALG; with HA | (13); (14); (15) |
| | **Alginate (ALG)** | In vitro encapsulation of isolated ovarian cells and preantral follicles to assess cell viability and ECM support; followed by in vivo pilot grafts for biocompatibility | Biocompatible; mimics ovarian ECM; supports 3D cell organization | Limited stability; Matrigel variability; short culture period | Develop fully defined, synthetic ECM analogues for extended in vitro culture and translational use | Preantral & antral | Mice | With Matrigel | (7) |
| | **Hyaluronan (HA) tyramine-linked hydrogel** | 3D culture of preantral mouse follicles; MII oocytes obtained; vitrification protocol compatible | ECM-like; tunable viscoelasticity; in-gel ovulation/maturation | Mainly mouse validation; long culture times | Extend to human follicles; growth-factor integration | From Preantral to antral | Mouse | Enzymatic crosslinking in-gel | (16); (17) |
| | **Collagen** | Widely used for follicle encapsulation/ culture**;** (often as pre-culture before grafting) | Highly biocompatible; promotes adhesion & remodeling | Rapid degradation (needs reinforcement for implants) | Collagen-based composites for stability | Preantral & antral | Mice | - | (18) |
| *Natural ECM-derived scaffolds* | **Decellularized ovarian ECM** | In vitro folliculogenesis and tissue culture on ovary-specific ECM | Tissue-matched cues; supports survival, proliferation, steroidogenesis | Low standardization for scale-up | Scalable decell & GMP matrices | Cortical ECM (follicle niche-derived) | Mice, porcine, bovine, human | - | (19); (20); (21); (22) |
| *Electrospun fiber scaffolds* | **Electrospun PCL (patterned/random)** | Long-term in vitro follicle culture (ovine, porcine, mouse); bioinspired 3D support | Highly biocompatible; scalable; topologically tunable | Equipment/certification costs; translation to implants pending | Toward transplantable AO; integrate cues/angiogenic factors | From Preantral to early antral, antral | Ovine, porcine | With gonadotropins, GFs, OSE co-culture | (23–25); (26); (27) |

## References


1. Paulini F, Vilela JMV, Chiti MC, Donnez J, Jadoul P, Dolmans MM, et al. Survival and growth of human preantral follicles after cryopreservation of ovarian tissue, follicle isolation and short-term xenografting. Reprod Biomed Online. 2016 Sep 1;33(3):425–32.
2. Smith RM, Shikanov A, Kniazeva E, Ramadurai D, Woodruff TK, Shea LD. Fibrin-mediated delivery of an ovarian follicle pool in a mouse model of infertility. Tissue Eng Part A [Internet]. 2014 Nov 1 [cited 2024 Jan 27];20(21–22):3021–30. Available from: https://pubmed.ncbi.nlm.nih.gov/24802617/
3. Kniazeva E, Hardy AN, Boukaidi SA, Woodruff TK, Jeruss JS, Shea LD. Primordial Follicle Transplantation within Designer Biomaterial Grafts Produce Live Births in a Mouse Infertility Model. Sci Rep. 2015 Dec 3;5.
4. Rajabzadeh AR, Eimani H, Mohseni Koochesfahani H, Shahvardi AH, Fathi R. Morphological study of isolated ovarian preantral follicles using fibrin gel plus platelet lysate after subcutaneous transplantation. Cell J. 2015;17(1):145–52.
5. Kim J, Perez AS, Claflin J, David A, Zhou H, Shikanov A. Synthetic hydrogel supports the function and regeneration of artificial ovarian tissue in mice. NPJ Regen Med [Internet]. 2016 Dec 8 [cited 2025 Jan 9];1(1). Available from: https://pubmed.ncbi.nlm.nih.gov/28856012/
6. Gosden RG. Restitution of fertility in sterilized mice by transferring primordial ovarian follicles. Hum Reprod. 1990;5(5):499–504.
7. Vanacker J, Luyckx V, Dolmans MM, Des Rieux A, Jaeger J, Van Langendonckt A, et al. Transplantation of an alginate-matrigel matrix containing isolated ovarian cells: first step in developing a biodegradable scaffold to transplant isolated preantral follicles and ovarian cells. Biomaterials. 2012 Sep;33(26):6079–85.
8. Vanacker J, Dolmans MM, Luyckx V, Donnez J, Amorim CA. First transplantation of isolated murine follicles in alginate. Regenerative Med. 2014 Sep 1;9(5):609–19.
9. Tajbakhsh F, Tavana S, Kazemi Ashtiani M, Moini A, Amorim CA, Fathi R. Wharton's Jelly Hydrogel: An Innovative Artificial Ovary for Xenotransplantation of Isolated Human Ovarian Follicles. Biology (Basel). 2025 Oct 1;14(10):1340.
10. Laronda MM, Jakus AE, Whelan KA, Wertheim JA, Shah RN, Woodruff TK. Initiation of puberty in mice following decellularized ovary transplant. Biomaterials. 2015;50(1):20–9.
11. Liu WY, Lin SG, Zhuo RY, Xie YY, Pan W, Lin XF, et al. Xenogeneic Decellularized Scaffold: A Novel Platform for Ovary Regeneration. Tissue Eng Part C Methods. 2017 Feb 1;23(2):61–71.
12. Laronda MM, Rutz AL, Xiao S, Whelan KA, Duncan FE, Roth EW, et al. A bioprosthetic ovary created using 3D printed microporous scaffolds restores ovarian function in sterilized mice. Nat Commun. 2017;8(May):1–10.
13. Brito IR, Silva GM, Sales AD, Lobo CH, Rodrigues GQ, Sousa RF, et al. Fibrin-alginate hydrogel supports steroidogenesis, in vitro maturation of oocytes and parthenotes production from caprine preantral follicles cultured in group. Reprod Domest Anim. 2016 Dec 1;51(6):997–1009.
14. Shikanov A, Xu M, Woodruff TK, Shea LD. A Method for Ovarian Follicle Encapsulation and Culture in a Proteolytically Degradable 3 Dimensional System. Journal of Visualized Experiments. 2011 Mar 15;(49).
15. Shikanov A, Xu M, Woodruff TK, Shea LD. Interpenetrating fibrin-alginate matrices for in vitro ovarian follicle development. Biomaterials [Internet]. 2009 Oct [cited 2022 Nov 10];30(29):5476–85. Available from: https://pubmed.ncbi.nlm.nih.gov/19616843/

16. Desai N, Spangler M, Nanavaty V, Gishto A, Brown A. New hyaluronan-based biomatrix for 3-D follicle culture yields functionally competent oocytes. Reproductive Biology and Endocrinology. 2022 Oct 10;20(1):148.
17. Desai N, Attard M, Spangler M, Gishto A, Brown A, Wirtjes M. Application of a Novel Hyaluronan Hydrogel for Three-Dimensional Follicle Culture and Methodology for Mouse Ovarian Follicle Cryopreservation. Journal of Visualized Experiments. 2025 May 9;(219).
18. Telfer E, Torrance C, Gosden RG. Morphological study of cultured preantral ovarian follicles of mice after transplantation under the kidney capsule. J Reprod Fertil [Internet]. 1990 [cited 2025 Jan 9];89(2):565–71. Available from: https://pubmed.ncbi.nlm.nih.gov/2401983/
19. Mayorca-Guiliani AE, Willacy O, Madsen CD, Rafaeva M, Elisabeth Heumüller S, Bock F, et al. Decellularization and antibody staining of mouse tissues to map native extracellular matrix structures in 3D. Nat Protoc. 2019 Dec 1;14(12):3395–425.
20. Jakus AE, Laronda MM, Rashedi AS, Robinson CM, Lee C, Jordan SW, et al. "Tissue Papers" from Organ-Specific Decellularized Extracellular Matrices. Adv Funct Mater. 2017 Sep 13;27(3).
21. Hassanpour A, Talaei-Khozani T, Kargar-Abarghouei E, Razban V, Vojdani Z. Decellularized human ovarian scaffold based on a sodium lauryl ester sulfate (SLES)-treated protocol, as a natural three-dimensional scaffold for construction of bioengineered ovaries. Stem Cell Res Ther. 2018 Sep 26;9(1):252.
22. Pors SE, Ramløse M, Nikiforov D, Lundsgaard K, Cheng J, Yding Andersen C, et al. Initial steps in reconstruction of the human ovary: survival of pre-antral stage follicles in a decellularized human ovarian scaffold. Hum Reprod. 2019 Aug 1;34(8):1523–35.
23. Di Berardino C, Liverani L, Peserico A, Capacchietti G, Russo V, Bernabò N, et al. When Electrospun Fiber Support Matters: In Vitro Ovine Long-Term Folliculogenesis on Poly (Epsilon Caprolactone) (PCL)-Patterned Fibers. Cells [Internet]. 2022 Jun 1 [cited 2022 Oct 5];11(12). Available from: https://pubmed.ncbi.nlm.nih.gov/35741097/
24. Peserico A, Di Berardino C, Capacchietti G, Camerano Spelta Rapini C, Liverani L, Boccaccini AR, et al. IVM Advances for Early Antral Follicle-Enclosed Oocytes Coupling Reproductive Tissue Engineering to Inductive Influences of Human Chorionic Gonadotropin and Ovarian Surface Epithelium Coculture. Int J Mol Sci [Internet]. 2023 Apr 1 [cited 2024 Jan 19];24(7). Available from: /pmc/articles/PMC10095509/
25. Di Berardino C, Peserico A, Camerano Spelta Rapini C, Liverani L, Capacchietti G, Russo V, et al. Bioengineered 3D ovarian model for long-term multiple development of preantral follicle: bridging the gap for poly(ε-caprolactone) (PCL)-based scaffold reproductive applications. Reprod Biol Endocrinol [Internet]. 2024 Dec 1 [cited 2024 Nov 22];22(1). Available from: https://pubmed.ncbi.nlm.nih.gov/39095895/
26. Fattahi A, Liverani L, Dittrich R, Hoffmann I, Boccaccini AR, Beckmann MW, et al. Optimization of Porcine Ovarian Follicle Isolation Methods for Better Developmental Potential. Tissue Eng Part A. 2020 Jul 1;26(13–14):712–9.
27. Liverani L, Raffel N, Fattahi A, Preis A, Hoffmann I, Boccaccini AR, et al. Electrospun patterned porous scaffolds for the support of ovarian follicles growth: a feasibility study. Scientific Reports 2019 9:1 [Internet]. 2019 Feb 4 [cited 2022 Nov 9];9(1):1–14. Available from: https://www.nature.com/articles/s41598-018-37640-1
